# How do microalgae perceive light in a high-rate pond? Towards more realistic Lagrangian experiments

**DOI:** 10.1098/rsos.180523

**Published:** 2018-05-30

**Authors:** David Demory, Charlotte Combe, Philipp Hartmann, Amélie Talec, Eric Pruvost, Raouf Hamouda, Fabien Souillé, Pierre-Olivier Lamare, Marie-Odile Bristeau, Jacques Sainte-Marie, Sophie Rabouille, Francis Mairet, Antoine Sciandra, Olivier Bernard

**Affiliations:** 1 Sorbonne Université, UPMC Univ. Paris 06, UMR 7093, LOV, Observatoire Océanologique, 06230 Villefranche-sur-mer, France; 2 Université Côte d'Azur, BIOCORE, INRIA, BP93, 06902 Sophia-Antipolis Cedex, France; 3 CNRS, UMR 7093, LOV, Observatoire Océanologique, 06230 Villefranche-sur-mer, France; 4 INRIA Paris, Team Ange, 2 rue Simone Iff, CS 42112, 75589 Paris Cedex 12, France; 5 IFREMER, PBA, Nantes 44311, France

**Keywords:** photosynthesis, hydrodynamics, modelling, photoacclimation, *Dunaliella salina*, non-photochemical quenching

## Abstract

Hydrodynamics in a high-rate production reactor for microalgae cultivation affects the light history perceived by cells. The interplay between cell movement and medium turbidity leads to a complex light pattern, whose forcing effects on photosynthesis and photoacclimation dynamics are non-trivial. Hydrodynamics of high density algal ponds mixed by a paddle wheel has been studied recently, although the focus has never been on describing its impact on photosynthetic growth efficiency. In this multidisciplinary downscaling study, we first reconstructed single cell trajectories in an open raceway using an original hydrodynamical model offering a powerful discretization of the Navier–Stokes equations tailored to systems with free surfaces. The trajectory of a particular cell was selected and the associated high-frequency light pattern was computed. This light pattern was then experimentally reproduced in an Arduino-driven computer controlled cultivation system with a low density *Dunaliella salina* culture. The effect on growth and pigment content was recorded for various frequencies of the light pattern, by setting different paddle wheel velocities. Results show that the frequency of this realistic signal plays a decisive role in the dynamics of photosynthesis, thus revealing an unexpected photosynthetic response compared to that recorded under the on/off signals usually used in the literature. Indeed, the light received by a single cell contains signals from low to high frequencies that nonlinearly interact with the photosynthesis process and differentially stimulate the various time scales associated with photoacclimation and energy dissipation. This study highlights the need for experiments with more realistic light stimuli to better understand microalgal growth at high cell densities. An experimental protocol is also proposed, with simple, yet more realistic, step functions for light fluctuations.

## 1. Introduction

Over recent years, biotechnological applications of microalgae have increased, particularly in the fields of cosmetics, pharmaceuticals and agrofood [1]. The main driver motivating these developments was the potential of some microalgae species in green chemistry, and especially for supporting large scale biofuel production [2]. This emerging approach is also characterized by reduced environmental impacts and enhanced productivity compared to terrestrial plants [3]. Indeed, the ability of these microorganisms to grow at high rates and to store large amounts of lipids has motivated substantial research activities over the last decade [4].

The ratio between the energy produced and the total energy involved is a key criterion when producing biofuel. The higher this ratio, the lower the costs and environmental footprint. As energy consumption emits greenhouse gases, the reduction of the energy demand remains a crucial challenge for sustainable microalgae cultivation [5]. Energy is required at different stages of the microalgal production process (growth, harvesting, post-treatment), with a significant demand for mixing processes during the microalgal growth phase, to ensure homogeneity and access to light. Two different kinds of devices are used to grow microalgae at industrial scales: photobioreactors, which are closed systems with short optical paths that support high cell densities (from 1 to 10 g l^−1^), and more basic raceway ponds, mixed by a paddle wheel, with lower cell densities (from 0.1 to 1 g l^−1^). The latter are probably more economical and are more widespread for large-scale algae production. In this paper, we focus on this less costly system, which might be better adapted for biofuel and food production [6].

**Figure 1.**
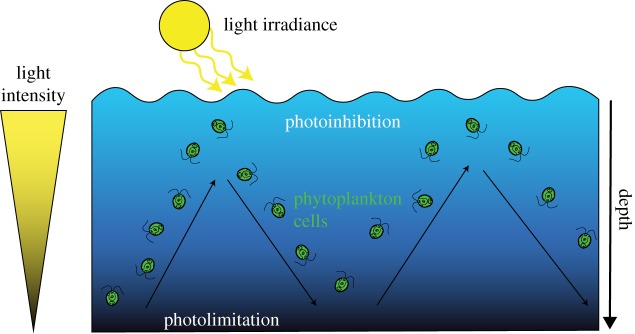
Schematic representation of an algal cell trajectory in an open pond raceway system.

The benefit of mixing has been extensively discussed in the literature to: (i) prevent cell sedimentation, (ii) ensure nutrient homogeneity, and (iii) facilitate CO_2_ transfer, while removing excess oxygen to limit photorespiration and oxidative stress [7]. Moreover, stirring also directly impacts light reception at the cell scale in a mixed turbid environment [8]. Focus on the cell scale shows that, due to water motion, cells receive a rapid succession of light spikes, reflecting the displacement of individual cells in the variable light field within the stirred culture system (figure 1).

The critical interplay of (i) light absorption and scattering at cell scale, (ii) cell density, and (iii) cell advection complicates the dynamics of the light received by a single cell. Furthermore, the way photons captured by the antennae are processed by the photosynthetic machinery is both nonlinear and dynamic [9,10]. The reaction centres excited by photons present significant de-excitation dynamics. If they receive additional photons while still being excited, they get damaged, leading to a decrease in efficiency (photoinhibition). Cells moderate the damaging impacts of high light (photoacclimation) by modulating their pigment content [11,12]. In particular, the synthesis of carotenoids involved in the xanthophyll cycle enhances a thermal dissipation of excess energy [13]. These so-called non-photochemical quenching (NPQ) mechanisms are dynamic and strongly influenced by the ratio of high to low light [14].

The impact of high frequency light fluctuations on both photosynthesis and photoacclimation is not fully understood, but appears to play an important role and could be used to enhance productivity. It has been studied by many authors [14–24] on the basis of a simple on/off experimental light signal. These works describe how the signal frequency affects photosynthesis: faster dark/light cycles increase photosynthetic efficiencies and enhance growth rates. Among these studies, Combe *et al*. [25] monitored the response of *Dunaliella salina* to simple light/dark cycles of increasing frequency. They used the same strain in the same experimental set-up (see §3), resulting in the typical response presented in electronic supplementary material, figure SI.2.1. Improvement in photosynthetic energy conversion in response to higher flash frequency is often called the ‘flashing light effect’ [15,26–28]. The work of Zarmi *et al*. [29] highlighted the dynamic mechanisms involved in photon harvesting and use, represented with mathematical models that consider the damaging and repair mechanisms of some key proteins in the photosynthetic apparatus. Such models explain the yield increase at higher frequencies, especially at high light.

Most of these works focused on photobioreactors with time scales much shorter than a second. In a raceway, light variations are slower and the commensurate acclimation processes have not been well studied [25]. There is, however, an underlying inconsistency in the literature dealing with photobioreactors and raceways. While numerous studies provide converging evidence for an enhanced photosynthetic efficiency under higher frequency of on/off signals [8,30], there is no clear experimental evidence that more mixing actually enhances growth. Qiang & Richmond [31] and Sobczuk *et al*. [32] studied different agitation velocities of a photobioreactor and identified an optimal agitation rate for a maximum cell density, beyond which increased stirring negatively affected growth. To explain the decrease in productivity at higher agitation, the authors mentioned shear stress and mortality due to the bursting of small gas bubbles. Overall, studies demonstrating productivity enhancement through increasing mixing are scarce compared to studies restricted to the flashing effect only, and they are rarely supported by experimental evidences. To the best of our knowledge, no work in the literature was unambiguously reported to support this dogma for raceway ponds.

This paper investigates the reasons for the surprising mismatch between a theory apparently consistent with many laboratory-scale experiments and reality. To address this point, we first revisited the typical approaches based on light signals that are far from actual patterns resulting from paddle wheel velocity and light field. Overall, the correspondence between experimental light signals and light actually received by a cell has not been considered. The originality of the present work lies in the consideration of a more gradual light evolution congruent with the pond hydrodynamic properties. The objective of this study is therefore to identify the effect of the mixing rate on photosynthesis under realistic light pattern conditions.

Our multidisciplinary approach begins with a downscaling viewpoint. A realistic light signal was reconstructed, using a hydrodynamic model that simulates Lagrangian cell trajectories. These dynamic light patterns were then analysed. The so-called ‘paragon’ (a specific cell trajectory representative of an ensemble of possible trajectories) was identified to represent a realistic light signal. A microcontroller-platform-based experimental device was then developed to expose a low-density population of microalgae to this light signal. By maintaining cultures at a low density, any self-shading was limited, ensuring that all cells in the experimental vessel received similar light levels. The population behaviour was thus homogeneous and reflected the state of a single cell. The experiments were carried out on a sufficiently long timeframe so that photoacclimation could take place and its dynamics could be studied. The observed response turned out to be more complex than what has so far been reported in the literature. We then discuss the experimental strategies required to study photosynthesis in more realistic conditions, including more realistic light stimuli with simple step functions.

## 2. Reconstruction of dynamic light patterns using hydrodynamic modelling

### 2.1. The hydrodynamic model

The flow of an incompressible water body in a raceway is accurately described by the Navier–Stokes equations. However, for a raceway, the mathematical model should also include the air–water interface. Such free-surface systems are more difficult to model and incur additional computational costs. Hydrodynamics in a raceway pond have usually been investigated with the goal of predicting dead zones or to improve pond designs especially for lowering agitation costs [33,34]. However its impact on light access and biomass productivity still remains open to study. Often, models are built using commercial software such as Fluent® which offers automated schemes for three-dimensional resolution of the Navier–Stokes equations. Given the numerical difficulties and the computational cost to approximate the Navier–Stokes solutions with standard finite volume approach, especially in the presence of a free surface, we use a recent approach based on a more efficient discretization of the Navier–Stokes equations [35,36]. This multilayer model applies Galerkin-type approximations along the vertical axis of the Navier–Stokes system yielding a set of partial differential equations with hyperbolic features. The system was numerically solved by considering a two-dimensional triangular mesh of the ground surface, the layers along the water depth giving rise to the third spatial dimension, as illustrated in electronic supplementary material, figure SI.4.1. This multilayer discretization of the Navier–Stokes system with mass exchanges has demonstrated enhanced accuracy and stability [35,37].

Moreover, the multilayer model has been successfully verified against analytical solutions for hydrostatic Euler and Navier–Stokes systems with a free surface [38]. We also added a specific forcing term mimicking the effect of the paddle wheel [36]. The impact of the paddle wheel on the fluid was represented as a normalized force applied by the wheel's blades proportional to the square of the velocity at each point. Here, we used a three-dimensional extension [39] to the model presented by Bernard *et al*. [36].

**Figure 2.**
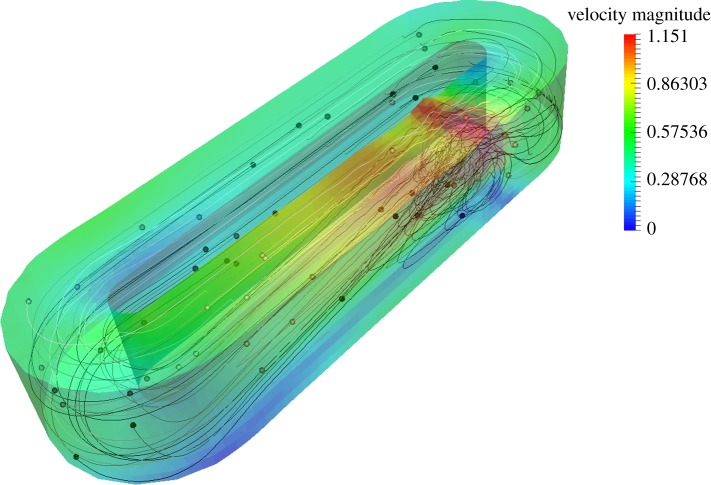
Numerical simulation of raceway hydrodynamics. Three-dimensional representation of the water volume and the velocity field (m s^−1^). Several particular trajectories are also shown.

A mesh was developed for the raceway pond in the Laboratory of Environmental Biotechnology (LBE, INRA Narbonne [40]) (electronic supplementary material, figure SI.4.1). The water-column depth (0.3 m) was divided into 20 horizontal layers. The simulation started with a static non-agitated medium, and the paddle wheel was instantaneously turned on. In electronic supplementary material, figure SI.4.2, we demonstrate that the computation has converged with this mesh (a more refined mesh has a marginal effect on results). In electronic supplementary material, SI.5, a momentum study was carried out, with computation of average depth and variance for 64 particles. It demonstrated ergodic properties for sufficiently long periods of time and showed that the numerical scheme conserved some structural properties of the trajectories. The raceway model was run for 4.2 h and the first hour of simulation was discarded in order to reach a stabilized regime. The velocity field together with a single cell trajectory is shown in figure 2.

### 2.2. Generation of Lagrangian trajectories and choice of a representative paragon

Lagrangian trajectories of 10 000 particles were reconstructed by integrating the velocity field for various initial particle positions. In addition, a Brownian motion model was applied to represent local diffusion effects [36].

**Figure 3.**
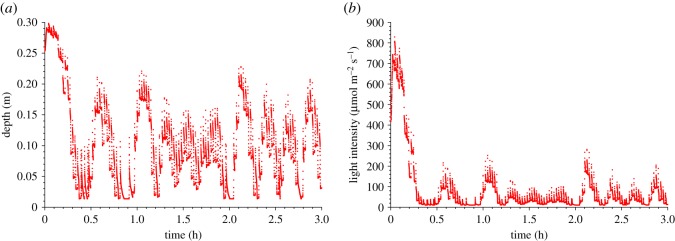
Depth (*a*) and light (*b*) pattern of the paragon trajectory.

The model was discretized into 20 layers. For each trajectory (after discarding the first hour), knowing its layer *i* at time *t*, a transition matrix was built by computing the probability for the particle to be at layer *j* at time *t *+ d*t*. A correspondence analysis was then carried out from this transition matrix. Based on the correspondence analysis results, we differentiated four sets of trajectories by performing a hierarchical ascendant clustering (Ward classification with Euclidian distances) to select the group containing particles that explore the entire water column. The trajectory closest to the centroid of the cluster was determined, i.e. the trajectory which minimized the following distance: $y_{j}=\text{min}\;\text{[ }{\left(A_{j}-\bar{A}\right)}^{2}\text{] }$ where *A_i_* is the probability matrix of trajectory *j*, and *Ā* is the average matrix probability of the particular group. The corresponding trajectory was called the paragon trajectory (figure 3) and represented a typical trajectory. The selected pattern was then cyclically repeated to obtain a periodic trajectory with a period *T* of 3.2 h. The light signal associated to this periodized trajectory was then considered.

Finally, the light received by the particle over time was computed, accounting for Beer–Lambert exponential attenuation:

2.1 $$I(t)=I_{0}\;\text{exp}(-kz),$$

where *I*_0_ is the light at the surface (here *I*_0_ = 850 µmol m^−2^ s^−1^), *k* is the light extinction coefficient, computed from *I*_L_, the light at the bottom of the raceway (assuming here *I*_L_ = 0.01 *I*_0_): $k=(1/L)\;\text{ln}\;(I_{0}/I_{L})$ and *z* is the depth of the particle at time *t*.

This light pattern shown in figure 3*b* was selected to be experimentally applied to the microalgal culture in a photobioreactor. Different mixing rates could have been simulated with the hydrodynamic model; however, comparing light patterns resulting from various hydrodynamic regimes is complex, and the choice of a specific trajectory associated with a given mixing rate complicates results interpretation. Instead, the effect of an increase or decrease in paddle wheel velocity was sketched by simply speeding up or slowing down the initial trajectory, assuming that the hydrodynamic regime was not structurally modified. This approach allowed us to focus on frequency effects. The initial periodized trajectory with period *T* of 3.2 h was then accelerated (considering periods *T*/3 and *T*/2) or slowed down (considering periods 2*T* and 3*T*). With this method, the daily light dose remained constant for all conditions.

This computational choice to reproduce several paddle wheel velocities facilitates comparison across experiments. The downside of this is that nonlinear effects in hydrodynamics with increased mixing are not represented, and would probably introduce biases in the light signal. Energy dissipation for various mixing regimes (and then different average fluid velocities) is presented in electronic supplementary material, figure SI.7.1.

## 3. Experimental set-up

The Chlorophyceae *Dunaliella salina* was grown axenically in f/2 medium within cylindrical double-walled glass photobioreactors with an effective capacity of 500 ml. Temperature was regulated at 27°C in the cultures. Magnetic stirrers homogenized the cultures. Bubbling was induced with air filtered through a Whatman filter. Experiments were carried out with duplicate fed-batch cultures diluted with fresh sterile medium every 8 days.

Photobioreactors were illuminated with arrays of six white light-emitting diodes (LEDs) installed on a circular aluminium frame. The LED drivers were controlled by an open-source electronics prototyping platform Arduino Mega 2560 to generate light patterns. Photosynthetically active radiation was monitored in each photobioreactor during the experiments using a 2π light sensor. Different light patterns were applied to the cultures, and an additional control was carried out using a continuous light of equal mean irradiance (82.2 µmol m^−2^ s^−1^).

The changes in algae population density and mean diameter were monitored twice-daily, in triplicate, using a Beckman Coulter counter. Pigments were extracted from algae with acetone and measured with a UV–visible spectrophotometer. Carbon and nitrogen cell quotas were determined with a CHN analyser after filtration onto pre-combusted GF/C filters (Whatman). See electronic supplementary material, SI.1, for details on the materials and methods.

## 4. Results

### 4.1. Growth rate

**Figure 4.**
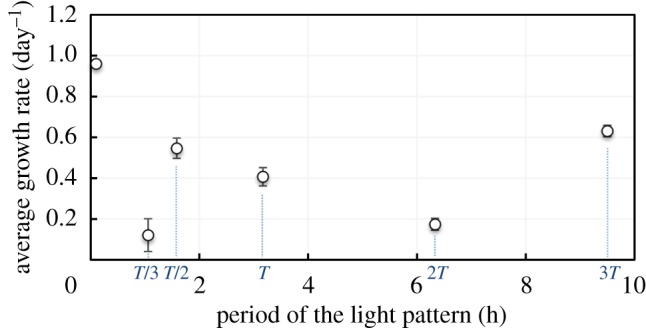
AGR as a function of light pattern period *T*. The point at datum *T* = 0 represents continuous illumination.

Average growth rates (AGR) for the various light patterns are represented in figure 4. In line with the experiment carried out with simple light/dark cycles [25] (see electronic supplementary material, figure SI.2), AGR obtained under continuous light is the highest. In particular, it is higher than that measured with the *T*-paragon trajectory by 136%. A light/dark signal with shorter period is known to stimulate photosynthesis and to induce higher growth rates [23,25]. A decrease in AGR with the signal period was observed from continuous illumination to 2*T*, except for the fastest *T/3*-light signal that surprisingly led to a very weak biomass growth. This experiment was repeated three times, but the results were eventually replicable, as illustrated in electronic supplementary material, figure SI.8.1. There was also an apparent paradoxical response for the signal with period 3*T* for which the growth rate was 55% higher than with the *T*-paragon, while, on the basis of the literature supported by simple light/dark cycles [14,22–25], this slowest signal was expected to be the least favourable to growth.

In agreement with the results of Combe *et al*. [25], intracellular carbon and nitrogen content did not significantly change across different conditions. Likewise, there was no significant difference in the C/N composition (*p* = 0.22) between light treatments (data not shown).

### 4.2. Acclimation mechanisms

**Figure 5.**
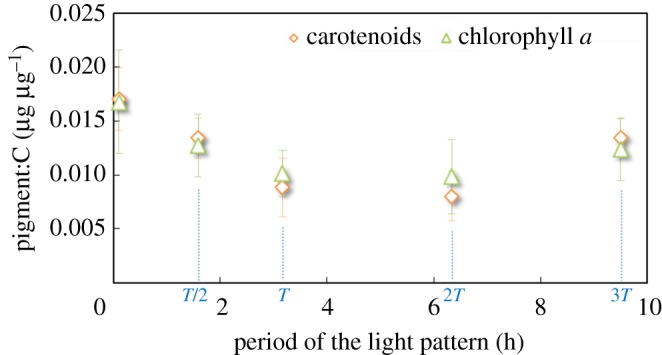
Chl *a*:C (µg µg^−1^) (green triangles) and Car:C (µg µg^−1^) (orange diamonds) of *Dunaliella salina* as a function of *T* (no measurement could be carried out for *T/*3 due to low biomass).

For realistic light signals, the levels of chlorophyll *a* (Chl:C) and carotenoids (Car:C) per carbon were affected by the changes in light pattern (figure 5). This observation is contrary to previous results obtained for higher (on/off) signal frequencies reported by Combe *et al*. [25]. Indeed, cells submitted to continuous light contained 65% more chlorophyll *a* and 36% more chlorophyll *b* per biomass unit compared to cells exposed to the paragon. Carotenoids followed a similar pattern: cells under continuous illumination contained 92% more carotenoids than the paragon, suggesting a different physiological reaction of cells depending on the light frequency. There was, however, no clear relationship between pigment content and the period of the light signal; it seems that a minimal pigment content was achieved for a light period in the range 3 to 6 h.

**Figure 6.**
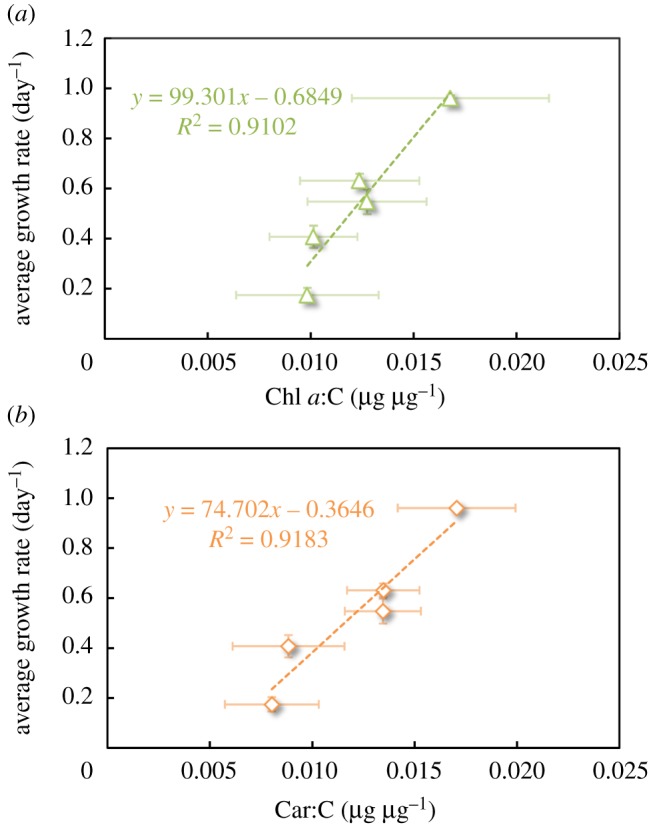
AGR (day^−1^) of *Dunaliella salina* as a function of (*a*) Chl *a*:C ratio (µg µg^−1^) and (*b*) Car:C ratio (µg µg^−1^) (no measurement could be carried out for *T/*3 due to low biomass).

The pigment content turns out to be strongly correlated with the growth rate (figure 6) (*R*^2 ^= 0.91, *n* = 5). This relationship is classical for chlorophyll, due to its central role in photosynthesis (at high light however, excess chlorophyll can trigger photoinhibition). Nevertheless, it was expected that an increase in photoprotectant pigments would decrease growth rate. Indeed, the role of carotenoids (mainly β-carotene for *D. salina*) is to dissipate excess energy and protect cells from free radicals [41]. At low light, while still dissipating light energy, carotenoids are expected to penalize growth. This drawback is only an advantage at high light intensities, by protecting the photosynthetic apparatus. As the light pattern was above 300 µmol m^−2^ s^−1^, for less than 10% of the time, it is expected that a high carotenoid content is detrimental to growth. Cells have therefore developed a dual strategy: simultaneously increase the chlorophyll content for enhancing photon capture (low-light acclimation) and increase NPQ for excess energy to be rapidly dissipated during the highest illumination periods (high-light acclimation). This analysis highlights a different type of acclimation strategy depending on the signal period. The most efficient strategy was a tendency toward higher chlorophyll and carotenoids

**Figure 7.**
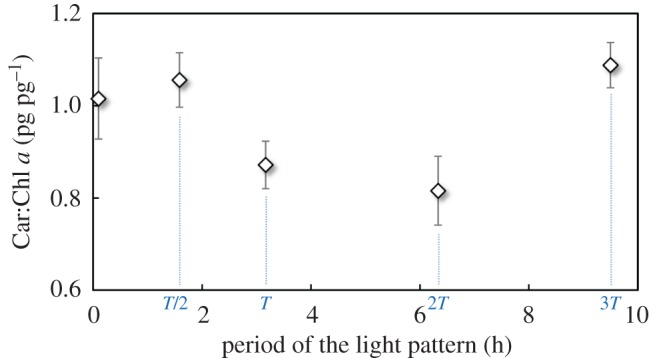
Car:Chl *a* ratio (pg pg^−1^) of *Dunaliella salina* as a function of *T* (no measurement could be carried out for *T/*3 due to the low biomass).

A closer look at the Car:Chl *a* ratio elucidates the cell response (figure 7). This ratio significantly changed (*p* < 0.05) with the light period, pointing out that each pigment class was regulated at a different rate. More precisely, figure 6 reveals that AGR increased faster with chlorophyll than with carotenoids. An increase in chlorophyll content was associated with higher carotenoids, and thus higher photoprotection, although growth rate was eventually enhanced. This consolidates the finding of Abu-Ghosh *et al*. [14], who demonstrated, with simpler light signals, that photosynthesis was enhanced when photochemistry and photoprotection were balanced. The xanthophyll cycles may perform differentially for dissipating energy especially during high light exposure. We therefore conjecture that this unexpected difference in cell photoacclimation partially explains the difference in growth rate and the apparent inconsistency with the previous results for simple light signals for which no marked change in photoacclimation was observed [25].

The Car:Chl ratio can be considered as a stress indicator [42]. Certain signal periods could probably induce further cell stress, resulting in significantly different growth rates. These observations need to be confirmed with additional experiments, but they highlight the role played by the photoacclimation dynamics. The mechanisms regulating carotenoid and chlorophyll syntheses have not been totally elucidated. The challenge now consists in discovering how the different light patterns trigger different photoacclimation strategies.

## 5. Discussion

### 5.1. What drives photoacclimation?

These results, when considering a realistic signal, reveal a more complex response than what has so far been reported under simple light patterns. The reconstructed trajectory is a complex dynamic signal comprising several time scales that are all potentially relevant to the different time scales of photosynthesis, photoprotection, and photoacclimation. The highest frequencies are associated to the Brownian motion added to the Lagrangian signal. At longer time scales, two key periodic events generate the observed dynamics. Firstly, each time a cell goes through the paddle wheel, it is rapidly advected to a different depth. The paddle wheel therefore clearly acts as the main process periodically redistributing the cell depth and thus actively contributing to distributing light access between the microalgae. When going through the paddle wheel, cells experience an abrupt light change over up to 12 s. This rather long period is due to a counter current beneath the paddle wheel that generates negative horizontal velocities. Hence, a cell can be trapped under the wheel for a certain amount of time. Secondly, and in addition to this phenomenon, cells travelling through the raceway bends also change depth, due to helicoidal flow triggered by the horizontal velocity gradient between faster trajectories along the external rim and slower, inner trajectories (see electronic supplementary material, SI.5). These two movements have a period of 1.5 min (average time to accomplish a full revolution around the pond). Apart from the period *T*, due to the cyclic repetition of the initial signal, the light signal contains a mix of subfrequencies, all of which may impact photosystem and photoacclimation dynamics. It is possible that some of these time scales influence the yield of photon harvesting and processing more intensively [43]. They also induce different responses in energy dissipation mechanisms. In particular, at high light, the xanthophyll cycle dissipates photons through non-photochemical quenching. For chlorophytes, the violaxanthin cycle plays a key role in NPQ, exerting a photoprotective effect through accumulation of zeaxanthin, which facilitates a thermal dissipation of the excess light energy. As demonstrated for *Dunaliella salina* by Abu-Ghosh *et al*. [14], this process is highly dynamic, and its efficiency is closely related to the light regime, in particular to the time ratio between high and low light. However, these acclimation mechanisms have generally been studied at short time scales (frequencies faster than 10 Hz) that occur in photobioreactors, whereas we are considering here slower processes typical of raceways dynamics. Modification of the xanthophyll pool is likely to be affected by light fluctuations at a scale of several hours as has been observed for higher plants [44]. By speeding up or slowing down the signal, all frequencies shift and some of them may eventually prove less (for the *T*/3 pattern) or more (the 3*T* pattern) efficient for photosynthesis. Beyond the signal frequency, the shape of the signal itself impacts photosynthesis. For a given frequency, schematic signals with different time ratios of high and low light affect growth rates [25]. However these inherent signal properties are not affected when lengthening or shortening the light signal, and more generally, neither the average light nor its standard deviation change.

A simple light/dark signal has not been shown to significantly affect photoacclimation [25], but the response was different in our study. This unexpected behaviour when a complex realistic signal is applied suggests nonlinear effects on photosynthesis efficiency. There is a need for more investigations to better understand and separate these effects. To do so, experiments must be carried out using simple light signals that inherently hold key structural properties. These features are driven by hydrodynamics, and they are likely to trigger the mechanisms underlying the responses observed under realistic signals. In the following section, we discuss how a simple light pattern can be designed for incorporating these central physical properties.

### 5.2. Can the light pattern be more realistically sketched?

#### 5.2.1. Light distribution in a perfectly mixed cultivation system

The primary difference between the light pattern tested here and those reported in the literature is the correspondence of light distribution with hydrodynamics. Most of the mechanically agitated systems yield a homogeneous cell distribution. Homogeneous particle distribution results from fluid incompressibility while cells have the same density as water. However, in some cases, cells may not be iso-distributed along the culturing device, where dead zones lead to local accumulation of sedimented particles, locally increasing the particle density toward the bottom of the reactor. This behaviour has been numerically simulated, and some authors [33,45,46] proposed advanced designs to generate fluid circulation avoiding this source of productivity loss. Equidistribution is therefore targeted and reached with an efficient mixing system.

Simulating Lagrangian cell trajectories that reflect this theoretical iso-distribution property is numerically challenging. The numerical schemes used to compute Lagrangian trajectories from Eulerian velocity fields do not maintain these theoretical properties. Especially, particles rebounding from the domain boundaries (free surface or bottom) may generate an artificial local particle accumulation. This often leads to non-homogeneous distributions that must be artificially corrected [8].

Assuming cell equidistribution yields a constant probability density function (PDF) associated with particle depth:

5.1 $$f_{z}=\frac{1}{L}.$$

The PDF for light intensity, assuming a simple Beer–Lambert exponential decrease (equation (2.1)) can be analytically derived [47]:

5.2 $$f_{I}(I)=-\frac{1}{\text{ln}(\eta )I},$$

where *η* denotes the remaining fraction of light at the bottom: *η* = *I*_L_/*I*_0_ where *I*_0_ is surface light intensity and *I*_L_ is the intensity at the bottom.

As a consequence, PDF is a hyperbolic function that depends on the fraction of light reaching the raceway bottom. The average light in the system is

5.3 $$\bar{I}=\int_{I_{L}}^{I_{0}}f_{I}(I)I\text{d}I=I_{0}\frac{1-\eta }{-\text{ln}\;\eta }.$$

The probability of a cell receiving a light intensity higher than *I*_s_ is

5.4 $$P(I>I_{s})=\int_{I_{s}}^{I_{0}}f_{I}(I)\text{d}I=\frac{\text{ln}\;I_{s}/I_{0}}{\text{ln}\;\eta }.$$

As an illustration, if 1% of the light can still be detected at the reactor bottom (*η *= 0.01), 50% of the reactor depth is illuminated with a light level larger than 10% of the surface light. More generally 50% of the reactor is illuminated with a light intensity larger than $\surd \eta I_{0}$, and *p*% of the reactor is illuminated with a light intensity greater than $\eta^{p/100}I_{0}$ (for example, for *η *= 0.01, 15% of the reactor is illuminated with more than 50% of the original flux).

#### 5.2.2. Exact discretization into two layers does not exist

In most studies focusing on the flashing effect, light is represented with an oversimplified on/off signal. We offer a better option to represent the light pattern, assuming that the light source switches between two intensities. In this framework, each intensity represents the average light in a two-layer discretization of the raceway. The challenge lies in the determination of the light intensities to be applied and of the switching frequency for the light to represent key properties of the light signal at cell scale in a raceway.

Considering an upper euphotic layer of depth *h* with average light intensity *Ī*_E_, and a lower dark layer with average light intensity *Ī*_D_, we seek for a realistic probability (or frequency, if a periodic light signal is required) for the cell to switch between these layers. Both layers are characterized by their average irradiance. A desirable property would be to reconstruct a light signal switching between *Ī*_E_ and *Ī*_D_ (i) with time proportional to the probability of a particle to be in each layer, (ii) with an average light intensity *Ī* for the overall light signal equal to the mean light intensity in the process, and (iii) with light intensities *Ī*_E_ and *Ī*_D_ in agreement with the average light distribution in each layer. Such a light pattern would ensure correspondence between the biological stimuli and the physics of the process.

We demonstrate (see the electronic supplementary material, SI.9) that such behaviour cannot be achieved for a discretized water depth. Indeed, the average light when switching between *Ī*_E_ and *Ī*_D_ at rate *p* (*p = h*/*L* is the probability to be in the euphotic layer) is not the expected average light, unless the lower layer has a zero thickness.

#### 5.2.3. Variance computation

Instead of searching for a framework representing the average light in each layer, we seek a criterion that plays a key role in the photosynthetic dynamics: the variance of the light signal. Indeed, light variance is also strongly constrained by medium homogeneity and by the light extinction coefficient. The theoretical light variance based on the PDF of *I* is (see electronic supplementary material, SI.10)

5.5 $$\nu =\int_{I_{0}}^{I_{L}}f_{I}(I){\left(I-\bar{I}\right)}^{2}\text{d}I=\bar{I}\left[I_{0}\frac{1+\eta }{2}-\bar{I}\right].$$

It is worth noting that light variance is independent of fluid velocity and depends only on the light gradient through *η*. This non-intuitive result implies that the light variance is not related to the hydrodynamic properties, which conveniently simplifies the design of a realistic signal.

Now, considering a simple on/off light pattern oscillating between *I*_0_ and 0 at frequency $p=\bar{I}/I_{0}$ (case A), a much larger variance $\left(\bar{I}(I_{0}-\bar{I})\right)$ is obtained than that given by equation (5.5). For example, for *η* = 1%, the variance induced by a light commutation between *I*_0_ and 0 is 2.7 times greater than the variance in the actual culture process.

We propose an alternative strategy to synthesize simple, yet realistic light signals for laboratory experiments. We relax constraint (iii) on the realism of *Ī*_E_ and *Ī*_D_, and, instead, impose a condition of light variance: (iii.b) variance of light intensity according to (5.5). In this new framework, *I*_E_ and *I*_D_ are no longer assumed to represent the average light intensity within each layer.

There are infinite combinations of *I*_E_ and *I*_D_ once *p* has been chosen (see electronic supplementary material, SI.11); in the following we consider two options: cells oscillating between *I*_E_ and darkness (case B) or between *I*_0_ and *I*_D_ (case C).

#### 5.2.4. Alternative choice for simple light patterns

**Case B. Switching between *I*_E_ and 0**

In this case, the upper light signal is *I*_E_, lower than the surface light *I*_0_. This approach is the simplest because it corresponds to an on/off signal. Here

5.6 $$I_{E}=I_{0}\frac{1+\eta }{2}\;\text{and}\;p=\frac{2\bar{I}}{I_{0}(1+\eta )}.$$

**Figure 8.**
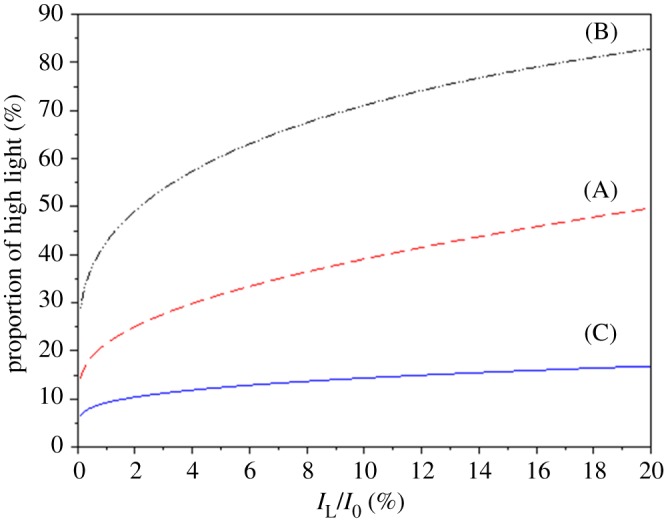
Proportion of high light (euphotic layer depth) with respect to *η *= *I*_L_/*I*_0_, for three different strategies. (A) The simple strategy for an on/off signal, with appropriate proportion of high-light *I*_0_, (B) light pattern with appropriate high-light fraction, average light, and standard deviation, with 0 as the lower light intensity (given by equation (5.6)), and (C) light pattern with appropriate high-light fraction, average light, and standard deviation, with *I*_0_ as the maximum light (given by equation (5.7)).

**Figure 9.**
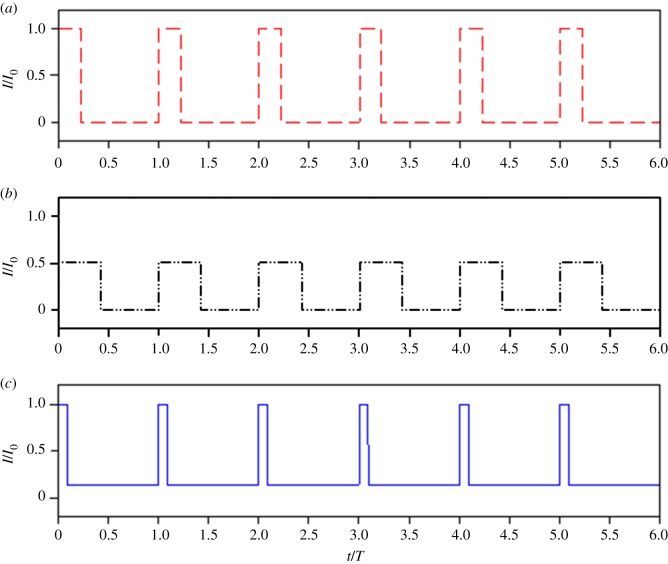
Representative step functions for light patterns in agreement with actual light distribution for *I*_L_/*I*_0 _= 0.01. Three possible strategies are presented: (*a*) the simple on/off strategy, with an appropriate proportion of high light *I*_0_, (*b*) light pattern with an appropriate high-light fraction, average light, and standard deviation, with 0 as lower light intensity (given by equation (5.6)), and (*c*) light pattern with an appropriate high-light fraction, average light, and standard deviation, with *I*_0_ as the maximum light (given by equation (5.7)) (appropriate for studying energy dissipation through the xanthophyll cycles).

The resulting light period is presented in figure 8 (case B), and the typical light pattern appears in figure 9*b*.

**Case C. Switching between *I*_0_ and *I*_D_**

Here, the upper light signal is *I*_0_. This approach is more relevant for experimental studies targeting photoinhibition and stimulating the xanthophyll cycle because it exposes cells to the highest light level they might encounter. In this option, *I*_D_ is not zero and can be computed (see electronic supplementary material, SI.11):

5.7 $$I_{D}=-\frac{{\left(1-\eta \right)}^{2}}{2(1-\eta +\text{ln}(\eta ))}I_{0}\;\text{and}\;p=\frac{\bar{I}-I_{D}}{I_{0}-I_{D}}.$$

The resulting light periods are presented in figure 8 (case C), and the typical light pattern is illustrated in figure 9*c*. It is noteworthy that this approach yields longer darker periods than what are generally studied in classical light/dark experiments (case A). In addition, the use of low light (and not zero) is more representative of real conditions. It is different from total darkness since it maintains a minimal photosynthetic activity and ensures a minimal level of oxygen in the cell.

Finally, we compare these step signals with the realistic paragon signal computed in §2.2 and experimentally applied to the culture. At first glance, the signal plotted in figure 3*b* is similar to the recommended step signal computed for case C. The time spent at high light for the paragon—i.e. with a light intensity higher than *p.I*_0_ (where *p* = 13.5%, as defined in case C)—is 15.8%, which is consistent with 9.2% of time at low light recommended for case C.

### 5.3. Toward realistic experiments

Lagrangian simulation is an appropriate route to downscale experiments studying the effects of mixing on the efficiency of photon use. Even though such a framework requires a complex experimental set-up and renders experiments more difficult to run, combining fast light variations (at the scale of seconds) with long-term experiments (at the scale of weeks) to account for cell photoacclimation remains of utmost importance. Better numerical schemes are expected to more accurately reconstruct the Lagrangian trajectory with higher Navier–Stokes fidelity. Also, advanced nonlinear optics describing light fields in a multidiffusive environment may more accurately represent light distribution. The key challenge to decipher the link between productivity and mixing energy is to reconstruct the light pattern for different mixing intensities. In electronic supplementary material, SI.7, we show that the dissipated energy is multiplied by 5.4 when the average fluid velocity is increased from 0.15 m s^−1^ to 0.3 m s^−1^. According to our experimental study, it is clear that this large increase in energy demand is not compensated by a larger productivity, and can even become counter-productive.

As demonstrated in this work, the Lagrangian patterns should have inherent theoretical properties (average light, standard deviation) independent of the hydrodynamic regime. To date, no numerical model guarantees these properties when considering various mixing regimes. Using relevantly designed light signals will thus contribute to better understand photosynthesis and NPQ in realistic conditions, provided that the high to low light exposure ratio is appropriately tuned to optical depth. Finally, it must be emphasized that simple step signals are generic and independent from the geometry of the process, while a Lagrangian-derived light pattern is tailored to a specific process, and associated with a mixing regime. Modifying the process geometry or the mixing intensity affects cell trajectories and eventually the light pattern. The Lagrangian trajectories should thus be recomputed for any change in the geometry. The cost for setting up the numerical simulation, and for adapting it to new conditions limits the approach.

## 6. Conclusion

Experiments usually performed with representative on/off light signals for studying the flashing effect in a dense culture are generally not consistent with the real physics. It is impossible to select an appropriate combination between two light intensities while respecting key physical principles. In this approach, the frequency of alternation between high and low light is related to both an underlying discretization into layers and the average fluid velocity. Reconstructing a realistic light pattern using a Lagrangian approach is, therefore, of importance for downscaling real processes. Using this approach, microalgae in laboratory environments experience similar conditions to those in industrial processes. Corresponding experiments growing *Dunaliella salina* revealed complex behaviours, different from previous on/off experiments, which cannot be explained by classical models. The trade-off between photosynthesis and photoprotection, through photoacclimation, seems to be triggered differently according to the light signal period. Further studies are needed to understand the intracellular regulation mechanisms that appear in these highly variable environments. The chlorophyll content proved to be highly dependent upon the rate of light variation, and thus on fluid velocity. If such a result is confirmed, there is an optimal agitation rate that can maximize growth. Experiments in high-density cultures could confirm these findings. This study highlights the benefits of a multidisciplinary approach, where improvement of the fluid dynamics modelling leads to a new generation of experiments emphasizing unexplored acclimation mechanisms.

## Supplementary Material

Experimental data

## Data accessibility

Data are available in a joined Excel file and are available from an open ftp repository (ftp://ftp-sop.inria.fr/biocore/OBERNARD/SuppInfo/).
